# Effect of Maturity Stage on Cereal and Leguminous Seeds’ Metabolome as Analyzed Using Gas Chromatography Mass-Spectrometry (GC-MS) and Chemometric Tools

**DOI:** 10.3390/metabo13020163

**Published:** 2023-01-23

**Authors:** Doaa B. Saied, Nehal S. Ramadan, Magdy M. El-Sayed, Mohamed A. Farag

**Affiliations:** 1 Chemistry Department, School of Sciences and Engineering, The American University in Cairo, New Cairo 11835, Egypt; 2 Chemistry of Tanning Materials and Leather Technology Department, National Research Centre, Giza 12622, Egypt; 3 Dairy Science Department, National Research Centre, Giza 12622, Egypt; 4 Pharmacognosy Department, Faculty of Pharmacy, Cairo University, Cairo 11562, Egypt

**Keywords:** legumes, cereals, chemometrics, GC–MS, seeds, maturity stage, metabolomics

## Abstract

Cereal and leguminous seeds are considered as major generic dietary source of energy, carbohydrates as well as proteins in the Mediterranean diet and are frequently consumed in **their** immature form in several regions including the Middle East. Hence, the current study aimed to assess metabolites’ heterogeneity amongst five major cereal and leguminous seeds of different species, and cultivars, i.e., *Triticum aestivum* L. (two cultivars), *Hordeum vulgare* L., *Vicia faba* L. and *Cicer arietinum* L., at different maturity stages. Gas chromatography mass-spectrometry (GC-MS) analysis using multivariate data analyses was employed for nutrient profiling and sample segregation assessed using chemometric tools, respectively. A total of 70 peaks belonging to sugars, fatty acids/esters, steroids, amino acids and organic acids were identified including sucrose, melibiose, glucose and fructose as major sugars, with butyl caprylate, hydroxybutanoic acid and malic acid contributing to the discrimination between seed species at different maturity stages. The investigation of total protein content revealed comparable protein levels amongst all examined seeds with the highest level detected at 20.1% *w*/*w* in mature fava bean. Results of this study provide a novel insight on cereal and leguminous seeds’ metabolomics in the context of their maturity stages for the first time in literature.

## 1. Introduction

Recently, switching to a plant-based diet is promoted as a viable option and a basis for better food sustainability, as well as enhanced health outcomes [1]. Ready-to-eat veggies taken from the very early stages of plant growth serve an important food, being enriched with bioactive metabolites, fitting with customer needs of health-based food [2]. For instance, immature seeds are known for being rich in dietary fiber; nevertheless, starch is more abundant in mature seeds, whereas fiber is scarce, suggestive for the use of immature seeds as a good alternative for different purposes and sensory attributes [3]. The stage of maturity, genotype as well as the complexity of food matrices are known to influence levels of bioactive compounds [4,5] warranting for the application of advanced analytical technologies for the assessment of their nutritive value and chemical composition [6].

Despite the complexity of global food security and enhanced nutrition and health issues, staple cereals as well as legumes represent an important dietary component in the food security topic [1,7]. Owing to their role in nitrogen fixation, legume crops are frequently cultivated in rotation alongside cereals [8]. The Mediterranean diet is known for being rich in legumes and whole seeds, with cereals included in the base of the nutritional food pyramid [9].

Cereal seeds account for up to 300 million tons yearly and hence are categorized amongst the world’s most important food pillars [10], particularly wheat and barley. Likewise, pulses, i.e., seed legumes, represent an essential pillar of diet worldwide and are cultivated as rain-fed crops on 95.7 million acres of land [11], among which broad beans as one of the oldest domesticated pulses and chickpeas are ranked first and third in their global production, respectively [8,12]. Seeds, worldwide, are mostly consumed in the mature dry state to extend their shelf life. However, in some regions such as Egypt, China, and Chile, immature seeds are consumed during their ripening season.

Cereal seeds pass through developmental stages, i.e., milk phase, dough phase, and ripening phase until harvesting, where each stage encompass four states, i.e., early milk, late milk, soft dough, late dough, grain hardening, etc. [13].

Wheat (*Triticum aestivum* L., Family: Poaceae) is recognized as a universal staple cereal crop [14]. From a nutritional aspect, Wheat accounts for over 20% of all dietary calories consumed worldwide, 37 percent of the total calories and 40 percent of protein in diets [15]. Wheat bioactive molecules are affected by the harvest period further, and its protein content is dependent on the seed maturity stage [16,17]. Whole wheat is rich in vitamins, minerals, dietary fibers and antioxidants [18], exemplified by phenolic acids viz. ferulic, vanillic, p-coumaric, caffeic and syringic acids. Wheat and its bran are reported to possess antioxidant activity as well as promote the reduced risk of chronic diseases viz. cardiovascular diseases and cancer [18,19]. On the other side, immature wheat seeds harvested at the milky phase are reported to be richer than mature ones in fibers, proteins, essential amino acids, oligosaccharides, particularly fructo-oligosaccharides, as well as soluble sugars, with a concomitant lack of immune-reactive gluten proteins, hence being suitable for celiac disease patients [20]. 

Barley (*Hordeum vulgare* L., Family: Poaceae) is a widely cultivated crop recognized as being amongst the most important cereals [21]. With regard to its chemical composition, barley is known for its richness in phenolics including flavonols, chalcones, flavones, proanthocyanidins, and flavanones [22]. Additionally, barley is reported as being rich in β-glucan, fiber, vitamin E, essential and non-essential amino acids [21,22]. Despite the underutilization of barley in nutrition, an increasing attention has been given towards barley owing to its potent health effects [22]. Nevertheless, the correlation between maturity stage and chemical composition in barley is not thoroughly studied [22].

Fava bean (*Vicia faba* L., Family: Fabaceae) is a legume cultivated in Egypt as one of its major producing countries [23]. Fava bean is regarded as a major source of essential nutrients viz. starch and protein (30% of lysine-rich) suggestive for its use as a major food [24]. Regarding its chemical composition, fava bean is rich in vitamin C, calcium, phosphorous, iron, zinc, polyphenols and γ-aminobutyric acid (GABA) [23]. In spite of all these nutrients, the existence of anti-nutritional factors, i.e., trypsin inhibitors, condensed tannins, phytic acid, saponins, lectins and favism-inducing factors viz. vicine and convicine, overshadows the nutritional value of fava beans [23]. With regards to green immature fava bean, it is reported to exhibit stronger antioxidant activity with a significantly higher phytochemical composition compared to mature ones [24], as the total phenolic content appears to decrease gradually along the maturation process [25]. Previous studies showed that immature seeds were enriched in monosaccharide sugars, while mature seeds were enriched with oligosaccharides, i.e., raffinose. 

Chickpea (*Cicer arietinum* L., Family: Fabaceae) is ranked second amongst pulse crops worldwide [26] regarded as a nutrient-dense food being rich in carbohydrates and proteins with a characteristic low fat content [27]. Chickpea is rich in proteins encompassing all essential amino acids. Fat content in chickpeas comprises almost 75% unsaturated fatty acids, being dominated by linoleic acid [28]. 

The selected studied specimens are consumed at both immature and mature stages; however, the mature dry form is more common due to the convenience of its storage and long shelf life. The green immature seeds are only consumed at their specified season prior to harvest time. Green chickpeas “malanah” are common in Upper Egypt, whereas green wheat “freekeh” is considered a main dish in the Mediterranean region; green bean pod is common in all Egypt. 

In order to address heterogeneity amongst specified seeds, i.e., (*Triticum aestivum* L., *Hordeum vulgare* L., *Cicer arietinum* L. and *Vicia faba* L.) in the context of their maturity stage, a metabolomics approach was applied for the first time in this study in a rather untargeted approach [29]. For metabolomics profiling, gas chromatography coupled to mass-spectrometry (GC/MS) is commonly adopted to characterize dietary sources’ nutrient profiles [29,30], and likewise in these mature and immature cereals and legumes. 

For better interpretation of such huge datasets, unsupervised multivariate data analyses are often adopted, e.g., principal component analysis (PCA), in addition to supervised methods, viz. orthogonal projection to least squares discriminant analysis (OPLS-DA), which can simplify metabolite data complexity and facilitate samples’ classification [30], and the identification of biomarkers.

## 2. Materials and Methods

### 2.1. Plant Samples

Mature and immature seeds of *Triticum aestivum* L. (two cultivars), *Hordeum vulgare* L., *Cicer arietinum* L. and *Vicia faba* L., were collected from El Qanater El Khayreya, Agricultural Experiment and Research Station, Faculty of Agriculture, Cairo University & Agricultural research station, Itay El-Barud, Egypt, respectively, as detailed in Table 1. 

### 2.2. Gas Chromatography-Mass Spectrometry (GC-MS) Primary Metabolite Profiling

#### 2.2.1. Samples’ Preparation and GC-MS Analysis Post Silylation

All seeds at different stages were freeze-dried using a lypophilizer and finely grinded to fine powder using an electric grinder. An exact weight of 20 mg of powdered samples was extracted with 1.5 mL 100% methanol containing 5 µL xylitol as an internal standard [31] followed by sonication for 30 min at 36 °C using Branson Ultrasonics, Carouge, SA Switzerland, then 15 min centrifugation (Universal centrifuge, Harmonic Series by Gemmy industrial®, Taipei, Taiwan) at 12,000× *g* to get rid of debris. Three technical replicates for each sample were analyzed under the same conditions to assess natural variation. Then, 100 μL of the methanol extract was left to evaporate till dryness in open screw-cap vials under a nitrogen gas stream. The dried methanol extract was then mixed and incubated with 100 μL of *N*-methyl-*N*-(trimethylsilyl)-trifluoroacetamide (MSTFA) previously (1:1) diluted with anhydrous pyridine (Yamato Scientific DGS400 Oven, Qte Technologies, Hanoi, Vietnam) for 45 min at 60 °C for derivatization prior to GC–MS analysis. Separation of silylated derivatives was completed on an Rtx-5MS column (Restek, Bellefonte, PA, USA) (30 m × 0.25 mm × 0.25 µm) fitted in a Schimadzu GC/MS/QP2010 (Kyoto, Japan) coupled to a SSQ7000 quadrupole mass spectrometer (Thermo-Fennigan, Breman, Germany). Primary metabolite analysis followed the exact protocol employed in [29].

#### 2.2.2. Identification of Metabolites and Multivariate Data Analysis of GC-MS Dataset

For identification, silylated metabolites were compared to n-alkanes (C20-C40) according to their retention indices (RI), Figure S1, and masses were matched to NIST spectral library database, and with standards whenever available. Peak deconvolution was first employed using AMDIS software (www.amdis.net) before mass spectral matching. Raw files are available at “GCMS raw files”. Peak abundance data were exported for multivariate data analysis by extraction using MS dial software (http://prime.psc.riken.jp/compms/msdial/main.html) with a retention time of 0–28 min, a mass range of 0–550 Da, and an accurate mass tolerance of 0.5 Da. Data normalization was performed to the amount of spiked internal standard, pareto-scaled and then subjected to principal component analysis (PCA), hierarchical clustering analysis (HCA) and partial least squares discriminant analysis (OPLS-DA) using SIMCA-P version 14.1 software (Umetrics, Sweden).

### 2.3. Total Protein Content

Total protein content in each sample was measured using the Leco^®^ protein analyzer (model no. fp528) by the Kjeldahl method [32] and the total nitrogen percentage was converted by factor multiplication (5.8×) to the protein percentage. All samples were measured in triplicate (*n* = 3), and expressed as mean ± SD. The data were analyzed using a one-way ANOVA (single factor) using Excel software. 

## 3. Results

### 3.1. Primary Metabolite Profiling viz. Sugars, Fatty and Organic Acids via GC-MS Post Silylation

**Figure 1:**
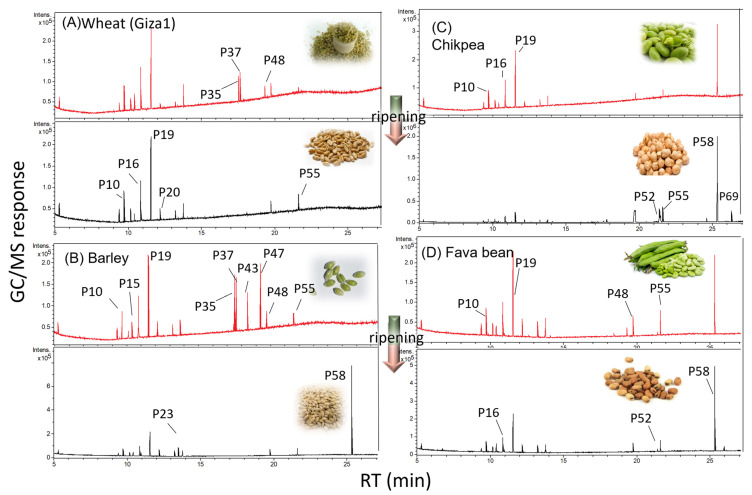
GC/MS chromatogram of silylated metabolites in (**A**) *T. aestivum* (cv. Giza1); (**B**) *H. vulgare* (cv. Giza 3); (**C**) *C. arietinum* (cv. Giza 1); (**D**) *V. faba* (cv. Sakha 3). Chromatograms are coded, where red is the immature stage, and black is the mature stage. Photos of samples are added in the top-right corner of each chromatogram. The corresponding metabolite number for each peak follows that listed in Table 2.

This study presents a comprehensive overview of the diverse primary metabolite profiles in five major edible seeds, i.e., cereals and legumes of selected species including wheat (cv. Gemeza11 and Giza1), barley (cv. Giza3), chickpea (cv. Giza1) & beans (cv.sakha3), in the context of their maturity stage as analyzed via GC-MS post-silylation (Figure 1). A total of 70 metabolites were identified (Table 2) including sugars, sugar alcohols, organic, amino and fatty acids, sterols, esters, and aromatics, as well as inorganic and nitrogenous compounds as detailed in the next subsections.

#### 3.1.1. Sugars and Sugar Alcohols

Sugars represent one of the most abundant metabolite classes in all studied seeds, amounting for 25–67% of the total metabolite content. Sugar content noticeably increased upon maturation in all seeds except in wheat and in accordance with reported increases in the sugar content of leguminous seeds upon maturation [33]. Among seeds, mature chickpeas (CM) showed the highest sugar level at ca. 67%.

In legumes, disaccharides were the most abundant sugars, represented by sucrose, (P58) and almost doubled upon maturation, accounting for ca. 16–22% in immature seeds and reaching ca. 31–35% upon maturation, followed by melibiose in chickpeas that increased from 7 to 30% upon maturation. Sucrose serves as a crucial signaling molecule that controls the production of crops, nitrogen fixation as well as seed filling [34,35,36]. Melibiose is a decomposition product of raffinose family oligosaccharides that exhibit potential immunostimulant and anti-allergic effects, in addition to being reported to improve mineral absorption and modulate gut microbiota [37]. The seeds most rich in melibiose include mature and immature chickpeas, posing them as good source of that valued sugar.

In cereals, mature seeds were similarly sucrose-enriched; nonetheless immature seeds were more enriched in monosaccharides viz. fructose (P34 and 37), and glucose (P43 and P47). The total sugar content in barley species almost doubled from 27 to 47% upon maturation.

On the contrary, wheat species showed an inconsistent pattern with legumes and barley. In wheat species (cv. Giza), a decline in sugars was observed, from 17 to 7% upon maturation, which is most probably attributed to total sugars’ reduction due to free sugars’ conversion into starch upon wheat maturation [20]. The immature wheat W2IM (cv. Gemeza11) exhibited almost comparable levels as the mature form W2M, highlighting certain metabolic process’ differences upon maturation within the same species of different cultivars. Similar to other seeds, sucrose increased in both mature forms of wheat regardless of the total sugar content variation.

Despite being represented by eight peaks as depicted in Table 2, sugar alcohols were present at much lower levels compared to cyclic sugars. They were mostly abundant in immature chickpea CIM at ca. 8.59%. Other seeds showed lower levels ranging from 2–4%, with the lowest found in wheat accessions. 

The difference in the sugar alcohols levels was only observed in chickpea seeds, as the maturation process resulted in a half reduction in CM to reach ca. 3.7%. CIM encompassed the highest pinitol (P39), and myo-inositol (P49) levels at 6 and 1%, respectively. In addition to their low caloric count, sugar alcohols exhibit multifunctional health-promoting qualities. For example, pinitol exerts antidiabetic [38], and nephroprotective [39] effects, whereas myo-inositol exert antidiabetic benefits [40].

#### 3.1.2. Fatty Acids/Esters and Steroids

Fatty acids, esters and steroids accounted for the second most abundant class after sugars, comprising ca. 15–32% of the total identified metabolites functioning as storage metabolites. Generally, their content was higher in BIM and CIM than their mature counterparts. Other seeds showed comparable levels at both stages, as seen in Table 2.

Butyl caprylate ester (P19) constituted the most abundant lipid in all seed specimens (ca. 2.8–13%) of the total detected metabolites, with wheat being most rich in this lipid without clear differences between maturity stages. The highest variation was observed in chickpea, bean, and barley, where immature seeds showed higher levels than mature ones. Butyl caprylate ester in CIM was at ca. 7.6% and dropped upon maturation to reach 2.9% followed by BEIM at ca. 10.7% to reach ca. 8.8% in BEM. Immature barley BIM encompassed 9.3% versus 6.3% in BM. Butyl caprylate ester is employed in food and beverage, pharmaceutical, and cosmetics industries, owing to its characteristic fruity flavor [41].

Saturated fatty acids were mostly predominated by palmitic acid (C16:0, P48) at ca. 2.7–6.7% and stearic acid (C18:0, P55) at ca. 2.6–5.7%. Palmitic acid in BIM was halved upon maturation, and in the case of stearic acid, it decreased in BM and CM compared to their immature seeds. Palmitic acid serves as a dietary energy source, though with conflicting evidence regarding its possible detrimental effects on health [42,43]. The advantage of plant-sourced palmitic acid over the animal-sourced one is well recognized, showing lower blood total cholesterol and low density lipoprotein cholesterol levels [44], whereas stearic acid provides firmness to fatty meals posing an excellent alternative to hydrogenation fats in food and cosmetics [40].

#### 3.1.3. Organic Acids

Total organic acids ranked third in abundance, comprising (7–29%) of the total identified metabolites, as shown in Table 2. In addition to their ability to preserve food, organic acids can lower food glycemic index, prolong the storage life of seeds, and improve digestion and protein consumption in animals [45].

Variation in total organic acids was detected in barley, bean, and chickpeas, where a major maturation-related reduction was evidenced in chickpeas detected at 6.9% in CM versus 17.5% in CIM. 

The most abundant organic acid was hydroxybutanoic acid and its isomer (P10 and P11), showing a decline upon maturation in all seeds, except in wheat, which showed an increase upon maturation.

Ketoisocaproic acid (P22), a metabolite of leucine [46] showed a decline in chickpea specimens upon maturation. Malic acid (P23), commonly used as a food acidulant for beverage enhancement [47], was detected at relatively high levels in BM at 5.5%, versus trace levels in BIM at 0.6%, likely accounting for the long shelf life of mature beans.

#### 3.1.4. Amino Acids

Legumes and cereals are reported as being protein-rich crops [48], especially wheat [49]. With regards to amino acids, wheat seeds represented by both cvs., were the richest in free amino acids, accounting for 13–16% of total metabolites, with levels found to be higher in mature specimens of Giza cultivar than immature ones. This was different to Gemeza cv., a wheat species that showed comparable results among the two stages, calling attention to certain cultivars’ variation in their metabolome response to maturation process.

In contrast, immature specimens of barley, bean and chickpea revealed higher content of amino acids at ca. 9.8–12.4% than mature ones, i.e., BM, BEM and CM, showing lower levels at ca. 3.5–10.2% and suggesting that the amino acid accumulation pattern upon maturation is seed specific. L-Glutamic acid (P16) and glycine (P9 and20) were the most common amino acids.

### 3.2. PCA and HCA Multivariate Data Analyses of Primary Silylated Metabolites of Mature and Immature Seeds of Different Cultivars

Two models of unsupervised-based pattern recognition were initially employed, including PCA and HCA, for the holistic assessment of the primary metabolite heterogeneity amongst mature and immature seeds of selected species. 

**Figure 2:**
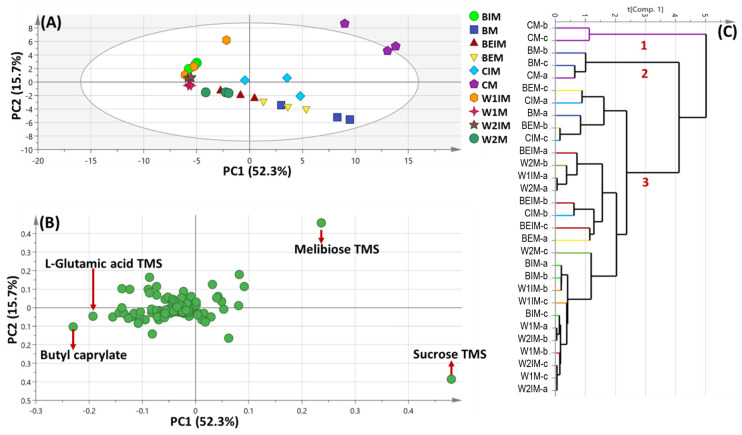
GC/MS-based unsupervised principal component analyses (PCA) and hierarchical clustering (HCA) of metabolites analyzed in whole sample dataset. (**A**) PCA score plot of PC1 against PC2. (**B**) Loading plot for PC1 and PC2, with contributing primary metabolites and their assignments. The metabolome clusters are located at the distinct positions in two-dimensional space described by two vectors of principal component 1 (PC1) = 52.3% and PC2 = 15.7%. (**C**) HCA plot showing 3 main subdivisions.

Principal component multivariate data analysis (PCA) was demonstrated by two orthogonal components, accounting for 68% of the total variance prescribed by PC1 and PC2 (Figure 2A). An obvious segregation between mature chickpea CM, mature barley BM and mature bean BEM specimens from others could be observed along PC1, clustered with positive score values (right side in PC1), whereas immature as well as mature wheat specimens were positioned in the middle and in left side along PC1 (negative score values). Examination of the loading plot (Figure 2B) revealed that sugars viz. melibiose contributed the most to mature chickpea CM seeds’ segregation, whereas sucrose was more abundant in mature barley BM. Glutamic acid as well as butyl caprylate were found to be the most enriched in mature wheat (cv. Giza1) W1M specimens. 

HCA showed a dendrogram of three distinct clusters (Figure 2C), where CM and BM were segregated in two distinct clusters. Wheat species of both mature and immature forms were aggregated in a subdivision of the same cluster, hence, HCA failed to characterize the impact of maturity stage on specified specimens based on their silylated primary metabolite composition.

### 3.3. OPLS-DA Analysis of Immature versus Mature Seeds Primary Silylated Metabolites in All Seed Specimens Dataset

OPLS-DA was further employed to assess seed discrimination based on maturity stage (mature and immature), for better segregation than observed in PCA analysis. Hence, a model of immature seeds (W1IM, W2IM, BIM, BEIM & CIM) against mature ones (W1M, W2M, BM, BEM, CM) in another class was first constructed (Supplementary Figure S2A). The OPLS model exhibited Q^2^ = 0.66 indicating the model predictability, and total variance coverage of 81.4% (R^2^ = 0.81). The respective loading S-plot (Supplementary Figure S2B) revealed that sucrose (peak 58) was enriched in mature seeds compared to immature ones, with a significant *p* value of 0.001.

### 3.4. Multivariate Data Analyses of the Primary Silylated Metabolites in Cereals (Wheat and Barley) Models

**Figure 3:**
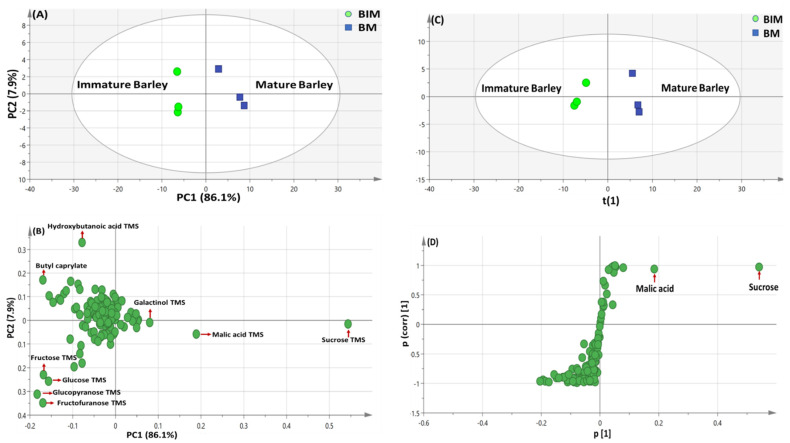
GC/MS-based principal component analyses (PCA) of metabolites analyzed in mature vs. immature *H. vulgare* (cv. Giza 3). (**A**) PCA score plot of PC1 against PC2. (**B**) Loading plot for PC1 and PC2, with contributing primary metabolites and their assignments. The metabolome clusters are located at the distinct positions in two-dimensional space described by two vectors of principal component 1 (PC1) = 86.1% and PC2 = 7.9%. (**C**) OPLS-DA score plot derived from modeling silylated primary metabolites of mature vs. immature *H. vulgare* specimens (sp. *Giza 3*) (*n* = 3). The respective loading S-plots (**D**) show the covariance p[1] against the correlation p(cor)[1] of the variables of the discriminating component of the OPLS-DA model with a *p*-value of 0.1. Designated variables are highlighted and identifications are discussed in the text.

**Figure 4:**
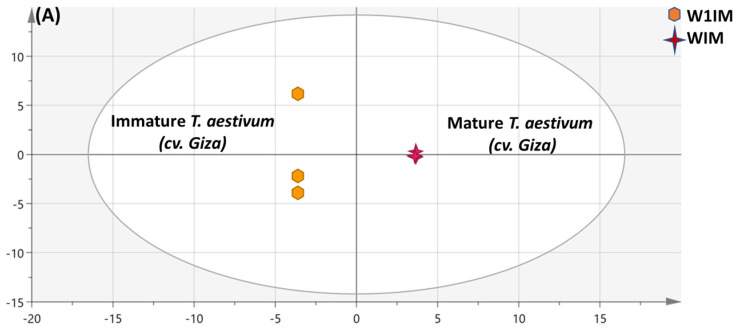

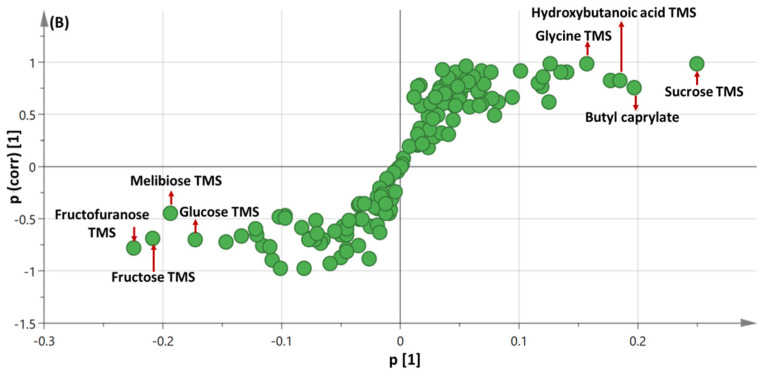
GC/MS dataset-based OPLS-DA score plot (**A**) derived from modeling silylated primary metabolites of mature vs. immature *T. aestivum* (cv. Giza) specimens (*n* = 3). The respective loading S-plot (**B**) shows the covariance p[1] against the correlation p(corr)[1] of the variables of the discriminating component of the OPLS-DA model, with a *p*-value of 0.2. Designated variables are highlighted and identifications are discussed in the text.

For better separation and to aid in identifying variation within each type of cereal, barley and wheat specimens were modeled individually one at a time (Figure 3, Figure 4 and Figure S3), as explained in the next subsections. 

#### 3.4.1. PCA and OPLS Multivariate Data Analyses of the Primary Silylated Metabolites in Barley Specimens

PCA model of barley seeds was illustrated by two orthogonal PCs, explaining 94% of the total variance, with distinct discrimination of BIM at the left side of PC1 separable from BM positioned on right side of PC1 (Figure 3A). The loading plot (Figure 3B) revealed that sugars viz. fructose, glucose as well as acid and ester viz. hydroxybutanoic acid & butyl caprylate, respectively found to be most enriched in BIM. In contrast, acid and sugar viz. malic acid & sucrose, respectively were more abundant in BM.

OPLS-DA supervised modeling of BIM against BM specimens (Figure 3C) was further attempted to confirm results derived from PCA model. The performance of the developed classification model was validated by the computed parameters “R^2^ (0.98)” and “Q^2^ (0.93)”, which showed improved prediction power than that of PCA model (Figure 3A). The observed segregation in the derived score plot (Figure 3D) was attributed to BM enrichment in malic acid (P23) & sucrose (P63), with though non-significant *p* value = 0.1.

#### 3.4.2. OPLS Multivariate Data Analyses of the Primary Silylated Metabolites in Mature vs. Immature Wheat Specimens

The PCA model employed for segregation of wheat specimens (W1IM, W1M, W2IM & W2M) revealed poor model fitness and predictability, and hence OPLS-DA score plot was performed to help identify variation within each cultivar separately one at a time (Figure 4 and Figure S3), respectively.

##### OPLS Multivariate Data Analysis of the Primary Silylated Metabolites in Mature vs. Immature *Triticum aestivum* (cv. Giza)

The impact of maturity on *T. aestivum* (cv. Giza) metabolites’ composition, which was failed to be explained in PCA analysis, was assessed by modeling W1IM against W1M via OPLS-DA (Figure 4A). The OPLS model exhibited stronger fitness and prediction power with R^2^ and Q^2^ values of 99.9% and 98.1%, respectively (Figure 4B). Sucrose (P63), butyl caprylate (P19), hydroxybutanoic acid (P10) and glycine (P9) dominated mature wheat (cv. Giza) metabolite composition. Fructose (P34 and 42), melibiose (P64), and glucose (P43 and P47), were more abundant in immature seeds though, with a non-significant *p* value of 0.2.

##### OPLS Multivariate Data Analysis of the Primary Silylated Metabolites in Mature vs. Immature *T. aestivum* (cv. Gemeza 11)

Likewise, to assess segregation of W2IM vs. W2M upon failure of separation in PCA, supervised OPLS-DA (Supplementary Figure S3A) was implemented. Model showed predictability Q^2^ = 93.7% and total variance coverage R^2^ = 99.2% of the score plot. The respective loading S-plot (Supplementary Figure S3B) revealed the enrichment of mature W2M with sucrose (P63) compared to immature specimens, while the latter was enriched in fructofuranose (P34) and fructose (P42), with a *p* value of 0.09, in accordance with what was observed in the previous section.

### 3.5. Multivariate Data Analyses of The Primary Silylated Metabolites in Legumes Models

**Figure 5:**
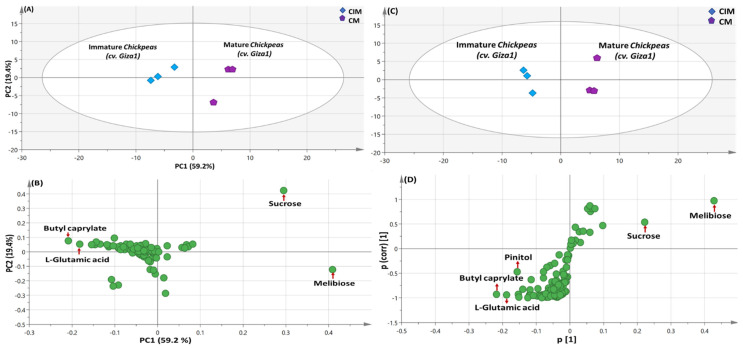
GC/MS-based PCA and OPLS analysis of metabolites analyzed in mature vs. immature *C. arietinum* (sp. Giza 1). (**A**) PCA score plot of PC1 against PC2. (**B**) Loading plot for PC1 and PC2, with contributing primary metabolites and their assignments. The metabolome clusters are located at the distinct positions in two-dimensional space described by two vectors of principal component 1 (PC1) = 59.2% and PC2 = 19.4%. (**C**) OPLS-DA score plot derived from modeling silylated primary metabolites of mature vs. immature *Cicer arietinum* (cv. *Giza 1*) *specimens* (*n* = 3). The respective loading S-plots (**D**) show the covariance p[1] against the correlation p(cor)[1] of the variables of the discriminating component of the OPLS-DA model. Cut-off values of *p* = 0.06 were used. Designated variables are highlighted and identifications are discussed in the text.

For detecting variation amongst each type of specified legumes and for improved discrimination, chickpea and bean specimens were each modeled independently (Figure 5 and Figure S4).

#### 3.5.1. PCA and OPLS Multivariate Data Analyses of the Primary Silylated Metabolites in Chickpea Specimens

PCA model was constructed (Figure 5A) using immature chickpea CIM and mature CM only, which explained 79% of the total variance. PCA score plot showed that CIM was distinguished due to its enrichment in butyl caprylate ester (P19) & glutamic acid (P16), (Figure 5B) presenting immature seeds as of better nutritional value. Whereas, CM segregated, being most abundant in sucrose (P58) & melibiose (P64). 

Similar results were observed in OPLS-DA score plot (Figure 5C) & its respective loading S-plot (Figure 5D) except for the observation of pinitol (P39) being characteristic for CIM specimen besides butyl caprylate (P19) & L-glutamic acid (P16). The enrichment of the sugar alcohol pinitol, exhibiting comparable effect to disaccharides in mature seeds poses CIM as a better option than mature ones with regards to sugar profile.

#### 3.5.2. OPLS Multivariate Data Analyses of the Primary Silylated Metabolites in Mature vs. Immature Bean Specimens

An OPLS model was constructed for modeling BEIM against BEM, (Supplementary Figure S4A) as a supervised model. The model showed one orthogonal component with R^2^ = 0.99 and Q^2^ = 0.96, suggestive for strong fitness and prediction power. Moreover, loading S-plot (Supplementary Figure S4B) revealed the enrichment of mature BEM in monooleoyl glycerol (P60) and sucrose (P63), whereas hydroxybutanoic acid (P10) was found to be more enriched in BEIM metabolite composition with a *p*-value of 0.06.

### 3.6. Seeds Total Protein Content

The main objective was to investigate whether maturation stage had any effect on total protein content in seeds being an important dietary component [50] measured as N% *w*/*w*. Comparable protein levels were observed among all examined seeds ranging from 10.55 *w*/*w* in BM to 20.1 *w*/*w* in BEM. The lowest total protein content was detected in barley BM at 10.55, versus the highest level being found in fava bean in both mature BEM and immature BEIM seeds at 20.1 and 19.6%, respectively. Values for nitrogen content and the total protein content after factor conversion are illustrated in Supplementary Table S1 and Supplementary Figure S5. No significant difference in protein content upon maturation for all seeds was observed (*p* > 0.05).

## 4. Conclusions

The compositional heterogeneity in the primary metabolome composition of different seed species grown in Egypt in the context of their maturity stage was investigated through a holistic untargeted GC–MS metabolomics approach for the first time. 

Results of GC-MS analysis post silylation detected various metabolites belonging to sugars, fatty acids, amino acids, esters, steroids and organic acids. Di and oligosaccharides increased in all seeds upon maturation, whilst immature seeds were enriched with monosaccharides, thus might result in a higher postprandial blood glucose level. Additionally, detection of the sugar alcohol pinitol in mature chickpeas adds to its antidiabetic effect. Detected total organic acids were halved upon maturation in barley and leguminous specimens. Fatty acids viz. palmitic and stearic acids were higher in immature barley and chickpeas. Total amino acid content decreased upon maturation in barley and legumes, with a contrasting pattern in wheat, whereas no change was observed in the Gemeza cv. And there was increase in the Giza cv. 

**Figure 6:**
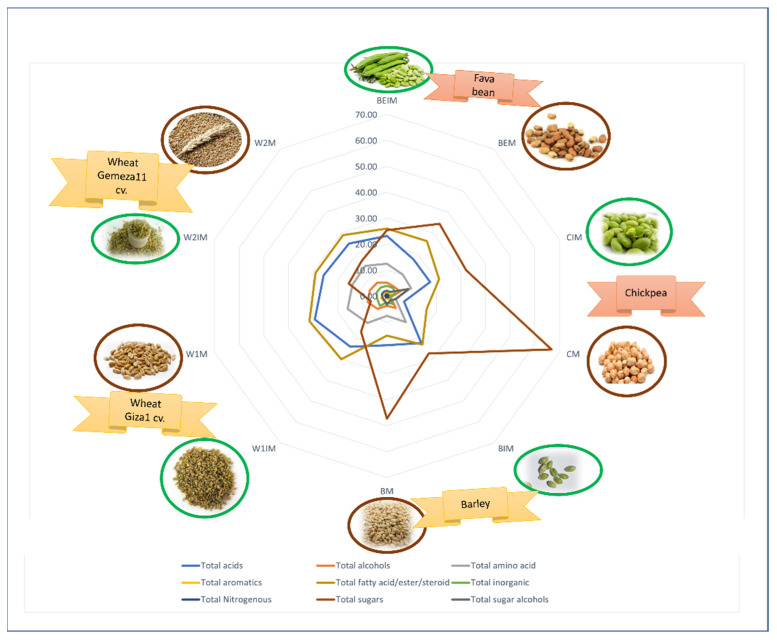
Radar plot of major metabolite classes contributing to the discrimination between investigated specimens, i.e., total acids, total aromatics, total nitrogenous, total alcohols, total fatty acid/ester, steroid, total sugars, total amino acids, total inorganic and total sugar alcohols, in investigated seeds. The figure demonstrates that total sugars are markers for CM and BM, total fatty acid/ester and steroids for BM and BIM, total acids for BIM, total amino acids for BIM, while total sugar alcohol is a marker for CIM. The corresponding sample codes follow those listed in Table 1.

Despite the lack of a specific maturation impact on metabolome among seeds, nevertheless barley showed certain similarities with leguminous seeds. While both wheat cultivars not only showed a contrasting pattern among the studied seeds, they also showed variation among each other, highlighting the variation among cultivars of same species. To sum up, a radar plot (Figure 6) was employed to reveal the relative abundances of metabolite classes contributing to the discrimination between the investigated specimens.

MVA of the primary metabolome revealed that sugars contributed to a common marker of maturation in all seeds. Major peaks viz. sucrose, melibiose, glucose, fructose, butyl caprylate, hydroxybutanoic acid, and malic acid distinguished between distinct seed species and offered new evidence for the metabolome of seeds at various stages of maturity.

Finally, the immature seeds presented improved lipid and amino acid profiles compared to their mature form, whereas mature seeds contributed less to high blood glucose levels. The total protein content assay did not reveal statistically significant differences upon maturation despite the amino acid differences, owing to the incorporation of all nitrogenous compounds in the adopted technique.

Such a hypothesis generated based on primary metabolites has yet to be confirmed based on monitoring changes in the secondary metabolome that are more likely to affect seeds’ health benefits, as well as using other techniques such as liquid chromatography coupled to mass spectrometry. Besides, authors recommend future studies to include more than two harvest points to conclude a general pattern of metabolome changes upon maturation.

## Figures and Tables

**Table 1:** Species, source, maturity stage and codes of the different seeds used in this study.

Name	Species	Family	Cultivar (cv.)	Source	Maturity Stage	Code
Wheat	*Triticum aestivum* L.	Poaceae	Giza	El Qanater ElKhayreya, Egypt	Immature	W1IM
					Mature	W1M
			Gemeza 11		Immature	W2IM
					Mature	W2M
Barley	*Hordeum vulgare* L.		Giza 3	Agricultural Experiment and Research Station, Faculty of Agriculture, Cairo University	Immature	BIM
					Mature	BM
Bean	*Vicia faba* L.	Fabaceae	Sakha 3	Agricultural Research station, Itay El-Barud, Egypt	Immature	BEIM
					Mature	BEM
Chickpea	*Cicer arietinum* L.		Giza 1		Immature	CIM
					Mature	CM

**Table 2:** Silylated primary metabolites expressed as the relative percentile of the total peak areas ± std. deviation, as analyzed using GC-MS from seeds at different maturity stages with results expressed as (*n* = 3). For codes, refer to Table 1.

Peak No.	Rt (min)	KI	Name	Barley		Bean		Chickpea		Wheat (cv. Giza)		Wheat (cv. Gemeza)	
				IM	M	IM	M	IM	M	IM	M	IM	M
Organic Acids													
1	6.74	1048	Lactic Acid (2 TMS)	1.55 ± 1.01	0.86 ± 0.30	0.89 ± 0.10	1.80 ± 0.66	0.79 ± 0.29	0.37 ± 0.02	1.16 ± 0.41	1.63 ± 0.22	2.06 ± 0.88	1.34 ± 0.31
6	7.01	1072	Glycolic acid (2 TMS)	0.35 ± 0.04	0.21 ± 0.09	0.35 ± 0.06	0.26 ± 0.05	0.26 ± 0.07	0.09 ± 0.01	0.36 ± 0.04	0.41 ± 0.06	0.37 ± 0.01	0.38 ± 0.06
7	7.50	1110	Oxalic acid (2 TMS)	0.52 ± 0.24	0.29 ± 0.09	0.72 ± 0.17	0.40 ± 0.18	0.36 ± 0.08	0.20 ± 0.04	0.54 ± 0.26	0.51 ± 0.03	0.42 ± 0.02	0.57 ± 0.18
8	8.2	1150	Hydracrylic acid (2 TMS)	0.23 ± 0.02	0.14 ± 0.03	0.24 ± 0.02	0.20 ± 0.03	0.19 ± 0.04	0.06 ± 0.01	0.25 ± 0.03	0.32 ± 0.02	0.26 ± 0.03	0.26 ± 0.03
10	9.74	1241	Hydroxybutanoic acid (2 TMS)	4.12 ± 0.46	2.47 ± 0.83	4.53 ± 0.21	3.10 ± 0.49	3.45 ± 1.09	1.11 ± 0.13	4.51 ± 0.79	6.18 ± 0.07	4.81 ± 0.33	4.92 ± 0.37
11	9.78	1245	Hydroxybutanoic acid isomer (2 TMS)	3.25 ± 2.31	2.11 ± 1.64	4.89 ± 0.27	0.73 ± 0.11	2.85 ± 2.27	0.85 ± 0.64	4.74 ± 0.85	6.54 ± 0.02	3.78 ± 2.39	5.26 ± 0.41
13	10.19	1270	Octanoic acid (2 TMS)	3.61 ± 0.87	2.24 ± 0.65	3.99 ± 0.49	3.22 ± 0.34	2.89 ± 1.12	1.37 ± 0.17	4.13 ± 0.35	4.86 ± 0.28	4.36 ± 0.28	3.97 ± 0.50
17	10.95	1320	Succinic acid (2 TMS)	1.27 ± 0.12	1.26 ± 0.12	1.35 ± 0.14	1.20 ± 0.23	1.27 ± 0.21	0.37 ± 0.05	1.39 ± 0.24	1.59 ± 0.12	1.59 ± 0.03	1.49 ± 0.07
18	11.31	1343	Glyceric acid (3 TMS)	1.13 ± 0.09	0.61 ± 0.17	1.14 ± 0.49	0.84 ± 0.04	1.05 ± 0.17	0.33 ± 0.07	1.22 ± 0.10	1.39 ± 0.04	1.29 ± 0.08	1.29 ± 0.10
22	13.25	1481	Ketoisocaproic acid (TMS)	2.66 ± 0.45	1.87 ± 0.44	2.96 ± 0.17	2.57 ± 0.27	2.32 ± 0.32	0.87 ± 0.09	2.40 ± 0.21	3.34 ± 0.12	3.13 ± 0.19	3.02 ± 0.07
23	13.51	1500	Malic acid (3 TMS)	0.62 ± 0.17	5.50 ± 1.65	0.33 ± 0.02	0.82 ± 0.16	0.40 ± 0.12	0.14 ± 0.03	0.53 ± 0.19	0.28 ± 0.04	0.77 ± 0.08	0.30 ± 0.05
24	13.74	1518	Unknown	0.73 ± 0.19	0.48 ± 0.13	0.69 ± 0.06	0.84 ± 0.13	0.63 ± 0.29	0.20 ± 0.02	0.79 ± 0.05	1.06 ± 0.15	0.95 ± 0.15	0.94 ± 0.18
26	16.05	1703	Suberic acid (2 TMS)	0.21 ± 0.03	0.12 ± 0.05	0.19 ± 0.09	0.20 ± 0.05	0.18 ± 0.07	0.06 ± 0.00	0.27 ± 0.04	0.27 ± 0.06	0.24 ± 0.05	0.20 ± 0.04
31	17.19	1800	Azelaic acid (2 TMS)	0.32 ± 0.06	0.17 ± 0.03	0.31 ± 0.02	0.25 ± 0.03	0.29 ± 0.07	0.17 ± 0.10	0.51 ± 0.19	0.35 ± 0.04	0.37 ± 0.03	0.39 ± 0.02
32	17.2	1801	Azelaic acid isomer (2 TMS)	0.48 ± 0.04	0.26 ± 0.07	0.42 ± 0.04	0.34 ± 0.07	0.40 ± 0.05	0.23 ± 0.11	0.64 ± 0.15	0.51 ± 0.07	0.49 ± 0.04	0.53 ± 0.06
36	17.58	1837	Citric acid (4 TMS)	1.41 ± 0.49	0.38 ± 0.11	0.16 ± 0.02	0.61 ± 0.17	0.09 ± 0.02	0.51 ± 0.22	0.74 ± 0.45	0.08 ± 0.02	0.85 ± 0.09	0.10 ± 0.02
Total organic acids				22.44	18.98	23.17	17.39	17.43	6.92	24.18	29.32	25.73	24.96
Alcohols													
3	5.31	918	Ethylene glycol (2 TMS)	2.61 ± 0.10	1.61 ± 0.50	2.80 ± 0.20	2.32 ± 0.40	2.13 ± 0.64	0.71 ± 0.08	2.80 ± 0.45	3.61 ± 0.06	3.03 ± 0.11	3.17 ± 0.26
5	6.63	1037	Propanediol (2 TMS)	0.47 ± 0.04	0.27 ± 0.09	0.49 ± 0.04	0.38 ± 0.05	0.39 ± 0.10	0.12 ± 0.01	0.48 ± 0.06	0.70 ± 0.03	0.50 ± 0.02	0.54 ± 0.07
14	10.42	1286	Glycerol (3 TMS)	2.63 ± 0.04	1.93 ± 0.38	1.89 ± 0.34	1.72 ± 0.52	2.85 ± 1.21	0.64 ± 0.14	2.90 ± 0.59	3.48 ± 0.36	3.46 ± 0.64	2.70 ± 0.17
Total alcohols				5.71	3.80	5.18	4.42	5.36	1.46	6.18	7.79	7.00	6.41
Amino Acids													
9	9.40	1219	Glycine (3 TMS)	2.03 ± 0.12	1.10 ± 0.46	2.23 ± 0.20	1.53± 0.37	1.49 ± 0.77	0.44 ± 0.03	2.35 ± 0.36	3.09 ± 0.15	2.41 ± 0.11	2.51 ± 0.27
16	10.88	1315	Glutamic acid (TMS)	6.50 ± 0.51	3.77 ± 1.25	6.67 ± 0.67	5.34 ± 0.79	5.41 ± 1.57	1.89 ± 0.21	7.64 ± 0.79	8.78 ± 0.47	7.46 ± 0.37	7.93 ± 0.63
20	12.19	1401	Glycine (3 TMS)/3-Aminoisobutyric acid (3 TMS)	3.01 ± 0.61	2.09 ± 0.48	2.55 ± 0.34	2.53 ± 0.37	2.21 ± 0.34	0.90 ± 0.13	1.97 ± 0.18	3.04 ± 0.12	3.17 ± 0.19	2.81 ± 0.21
21	12.63	1434	β-Alanine (3 TMS)	0.75 ± 0.04	0.48 ± 0.13	0.88 ± 0.13	0.68 ± 0.11	0.59 ± 0.09	0.20 ± 0.02	0.76 ± 0.07	1.04 ± 0.03	0.86 ± 0.05	1.01 ± 0.05
25	13.99	1536	Oxoproline (2 TMS)	0.16 ± 0.02	0.23 ± 0.06	0.07 ± 0.01	0.13 ± 0.02	0.08 ± 0.02	0.04 ± 0.01	0.13 ± 0.02	0.10 ± 0.03	0.16 ± 0.05	0.10 ± 0.00
Total amino acids				12.44	7.67	12.40	10.21	9.77	3.48	12.84	16.04	14.07	14.36
Aromatics													
4	6.56	1032	Phenol (TMS)	1.03 ± 0.06	0.63 ± 0.25	1.04 ± 0.11	0.85 ± 0.14	0.85 ± 0.22	0.19 ± 0.15	0.83 ± 0.52	1.43 ± 0.06	1.14 ± 0.08	1.22 ± 0.10
12	9.94	1254	Benzoic Acid (TMS)	0.99 ± 0.04	0.21 ± 0.08	0.30 ± 0.06	0.28 ± 0.05	1.01 ± 0.32	0.13 ± 0.05	1.48 ± 0.17	0.94 ± 0.07	0.47 ± 0.04	0.59 ± 0.04
Total aromatics				2.01	0.84	1.34	1.13	1.86	0.32	2.31	2.37	1.61	1.82
Fatty acid/ester/steroid													
19	11.58	1361	Butyl caprylate	9.28 ± 0.61	6.24 ± 2.10	10.74 ± 0.92	8.79 ± 1.45	7.57 ± 2.31	2.82 ± 0.36	10.63 ± 1.09	12.89 ± 0.52	11.94 ± 0.65	12.42 ± 1.01
38	17.72	1850	Myristic acid (TMS)	0.35 ± 0.03	0.18 ± 0.05	0.27 ± 0.01	0.26 ± 0.01	0.24 ± 0.07	0.09 ± 0.02	0.43 ± 0.08	0.36 ± 0.01	0.36 ± 0.01	0.34 ± 0.02
48	19.76	2045	Palmitic Acid (TMS)	5.15 ± 0.59	2.66 ± 1.00	3.99 ± 0.39	3.95 ± 0.29	3.75 ± 0.87	2.85 ± 3.07	6.62 ± 1.85	5.68 ± 0.42	5.35 ± 0.17	4.67 ± 0.11
50	20.72	2145	Margaric acid (TMS)	0.22 ± 0.02	0.15 ± 0.05	0.21 ± 0.03	0.20 ± 0.03	0.18 ± 0.05	0.12 ± 0.11	0.24 ± 0.02	0.30 ± 0.01	0.26 ± 0.03	0.25 ± 0.01
51	21.39	2215	Linoleic acid (TMS)	0.31 ± 0.04	0.49 ± 0.18	0.63 ± 0.23	0.90 ± 0.19	0.55 ± 0.31	0.82 ± 0.91	0.92 ± 0.09	0.93 ± 0.22	0.96 ± 0.03	0.92 ± 0.24
52	21.42	2218	Oleic Acid (TMS)	1.40 ± 0.17	1.08 ± 0.40	1.78 ± 0.33	2.38 ± 0.32	1.21 ± 0.04	2.53 ± 3.23	2.54 ± 0.59	2.12 ± 0.40	1.62 ± 0.36	1.97 ± 0.36
53	21.47	2224	Linoleic acid (TMS)	0.36 ± 0.02	0.11 ± 0.09	0.24 ± 0.35	0.41 ± 0.34	0.33 ± 0.21	0.39 ± 0.65	0.52 ± 0.35	0.15 ± 0.09	0.31 ± 0.05	0.13 ± 0.12
54	21.48	2225	Oleic Acid (TMS)	0.38 ± 0.04	0.20 ± 0.07	0.99 ± 0.58	1.33 ± 0.23	0.33 ± 0.08	0.87 ± 1.28	0.61 ± 0.20	0.83 ± 0.57	0.43 ± 0.04	0.41 ± 0.00
55	21.64	2243	Stearic acid (TMS)	4.23 ± 0.41	2.80 ± 0.89	4.68 ± 0.41	4.20 ± 0.31	3.59 ± 1.09	2.62 ± 2.32	4.89 ± 0.45	5.76 ± 0.17	5.29 ± 0.22	5.36 ± 0.28
56	24.36	2568	Palmitoylglycerol (2 TMS)	0.08 ± 0.02	0.06 ± 0.04	0.05 ± 0.04	0.07 ± 0.04	0.12 ± 0.04	0.11 ± 0.11	0.11 ± 0.01	0.10 ± 0.01	0.05 ± 0.03	0.07 ± 0.03
57	24.62	2601	1-Monopalmitin (2 TMS)	0.33 ± 0.09	0.38 ± 0.15	0.65 ± 0.09	0.42 ± 0.12	0.43 ± 0.14	0.56 ± 0.71	0.71 ± 0.12	0.76 ± 0.08	0.73 ± 0.05	0.71 ± 0.07
60	25.95	2768	Monooleoylglycerol (2 TMS)	0.26 ± 0.03	0.23 ± 0.05	0.43 ± 0.37	2.09 ± 0.34	0.25 ± 0.15	0.24 ± 0.28	0.30 ± 0.05	0.33 ± 0.06	0.32 ± 0.05	0.34 ± 0.11
61	26.09	2787	Glycerol monostearate (2 TMS)	0.22 ± 0.08	0.26 ± 0.10	0.44 ± 0.08	0.30 ± 0.10	0.29 ± 0.09	0.26 ± 0.25	0.47 ± 0.11	0.54 ± 0.10	0.48 ± 0.01	0.51 ± 0.05
65	26.33	2813	Sebacic acid (TMS)	0.34 ± 0.18	0.24 ± 0.13	0.58 ± 0.41	0.33 ± 0.17	2.02 ± 1.74	1.66 ± 0.44	0.61 ± 0.27	0.30 ± 0.18	0.42 ± 0.29	0.56 ± 0.07
66	26.46	2823	Lignoceric acid (TMS)	0.11 ± 0.01	0.08 ± 0.05	0.08 ± 0.01	0.09 ± 0.02	0.07 ± 0.01	0.08 ± 0.10	0.23 ± 0.14	0.14 ± 0.05	0.14 ± 0.03	0.09 ± 0.05
67	26.48	2826	Squalene	0.21 ± 0.05	0.13 ± 0.04	0.22 ± 0.08	0.47 ± 0.32	0.16 ± 0.04	0.08 ± 0.07	0.26 ± 0.05	0.29 ± 0.07	0.36 ± 0.12	0.20 ± 0.02
Total fatty acid/ester/steroid				23.21	15.29	25.99	26.18	21.09	16.10	30.09	31.47	29.00	28.96
Total inorganic compounds													
15	10.44	1286	Phosphoric acid (3 TMS)	3.81 ± 0.35	2.50 ± 0.40	3.90 ± 0.64	3.85 ± 1.06	3.26 ± 0.37	1.14 ± 0.23	4.72 ± 1.16	3.94 ± 0.15	4.23 ± 0.08	4.07 ± 0.05
	Total inorganic			3.81	2.50	3.90	3.85	3.26	1.14	4.72	3.94	4.23	4.07
Nitrogenous compounds													
2	5.24	913	Unknown	0.72 ± 0.10	0.41 ± 0.20	0.64 ± 0.14	0.53 ± 0.12	0.54 ± 0.14	0.16 ± 0.01	0.66 ± 0.14	0.64 ± 0.41	0.67 ± 0.07	0.68 ± 0.07
Total Nitrogenous compounds				0.72	0.41	0.64	0.53	0.54	0.16	0.66	0.64	0.67	0.68
Sugars													
33	17.37	1816	Methyl glucofuranoside (4 TMS)	0.20 ± 0.10	0.12 ± 0.08	0.22 ± 0.20	0.42 ± 0.25	0.32 ± 0.27	0.03 ± 0.02	0.39 ± 0.08	0.55 ± 0.39	0.50 ± 0.38	0.49 ± 0.20
34	17.47	1826	Fructofuranose (5 TMS)	1.86 ± 0.60	0.34 ± 0.14	0.15 ± 0.04	0.06 ± 0.02	0.11 ± 0.03	0.03 ± 0.01	0.84 ± 0.52	0.10 ± 0.01	1.04 ± 0.15	0.13 ± 0.02
35	17.56	1834	Fructofuranose isomer (5 TMS)	5.71 ± 1.92	0.96 ± 0.36	0.50 ± 0.03	0.99 ± 0.26	0.25 ± 0.08	0.85 ± 0.39	2.85 ± 1.71	0.19 ± 0.04	3.22 ± 0.36	0.25 ± 0.04
40	17.89	1865	Galactofuranose (5 TMS)	0.45 ± 0.16	0.07 ± 0.01	0.20 ± 0.04	0.04 ± 0.01	0.09 ± 0.04	0.04 ± 0.01	0.23 ± 0.06	0.07 ± 0.02	0.23 ± 0.02	0.07 ± 0.01
41	18.08	1883	Mannose (5 TMS)	0.13 ± 0.02	0.10 ± 0.01	0.46 ± 0.09	0.13 ± 0.08	0.09 ± 0.03	0.02 ± 0.01	0.12 ± 0.03	0.27 ± 0.18	0.20 ± 0.05	0.27 ± 0.18
42	18.4	1913	Fructose (5 TMS)	0.94 ± 0.15	0.33 ± 0.22	1.15 ± 0.08	0.04 ± 0.02	0.25 ± 0.16	0.02 ± 0.01	0.40 ± 0.26	0.09 ± 0.03	0.53 ± 0.11	0.20 ± 0.22
43	18.45	1917	Glucose (5 TMS)	4.57 ± 1.06	0.78 ± 0.27	1.29 ± 0.14	0.14 ± 0.04	0.34 ± 0.12	0.06 ± 0.02	1.51 ± 0.97	0.27 ± 0.14	1.74 ± 0.27	0.35 ± 0.06
44	18.57	1929	Galactopyranose, Mannose (5 TMS)	0.21 ± 0.06	0.13 ± 0.02	0.90 ± 0.23	0.07 ± 0.01	0.17 ± 0.05	0.02 ± 0.01	0.15 ± 0.06	0.05 ± 0.01	0.20 ± 0.01	0.06 ± 0.02
47	19.34	2002	Glucose isomer (5 TMS)	6.37 ± 1.57	1.14 ± 0.36	1.92 ± 0.20	0.21 ± 0.05	0.45 ± 0.16	0.08 ± 0.03	2.10 ± 1.44	0.35 ± 0.23	2.45 ± 0.34	0.47 ± 0.08
58	25.35	2693	Sucrose (8 TMS)	0.49 ± 0.21	41.21 ± 10.34	16.86 ± 4.92	31.18 ± 7.04	21.74 ± 10.20	35.22 ± 8.32	0.58 ± 0.21	3.26 ± 0.26	1.01 ± 0.16	12.54 ± 3.88
59	25.69	2737	Cellobiose, (isomer 2) (8 TMS)	0.05 ± 0.01	0.11 ± 0.10	0.19 ± 0.06	0.17 ± 0.13	0.10 ± 0.05	0.07 ± 0.03	0.35 ± 0.14	0.19 ± 0.14	0.20 ± 0.14	0.20 ± 0.11
62	26.24	2804	Lactose (8 TMS)	0.29 ± 0.3	0.31 ± 0.01	0.14 ± 0.13	0.13 ± 0.16	0.30 ± 0.49	0.01 ± 0.01	0.21 ± 0.22	0.11 ± 0.10	0.13 ± 0.14	0.21 ± 0.25
63	26.31	2810	Sucrose (8 TMS)	0.62 ± 0.05	0.43 ± 0.16	0.74 ± 0.07	0.61 ± 0.10	0.52 ± 0.13	0.21 ± 0.02	0.64 ± 0.07	0.82 ± 0.06	0.87 ± 0.08	0.89 ± 0.04
64	26.33	2812	Melibiose (8 TMS)	0.06 ± 0.01	0.03 ± 0.01	0.04 ± 0.01	0.04 ± 0.01	4.10 ± 5.24	4.06 ± 1.03	0.06 ± 0.01	0.03 ± 0.00	0.05 ± 0.03	0.07 ± 0.02
68	26.89	2863	Melibiose isomer I (8 TMS)	0.03 ± 0.02	0.06 ± 0.00	0.04 ± 0.01	0.03 ± 0.01	0.12 ± 0.05	0.61 ± 0.20	0.04 ± 0.03	0.03 ± 0.00	0.03 ± 0.00	0.03 ± 0.01
69	27.50	2918	Melibiose isomer II (8 TMS)	0.03 ± 0.00	0.15 ± 0.04	0.04 ± 0.01	0.07 ± 0.03	2.95 ± 1.66	25.33 ± 1.17	3.84 ± 6.59	0.05 ± 0.02	0.07 ± 0.01	0.23 ± 0.11
37	17.66	1843	Fructose (5 TMS)	5.38 ± 1.00	0.97 ± 0.40	0.51 ± 0.03	0.10 ± 0.01	0.19 ± 0.06	0.06 ± 0.01	2.76 ± 2.26	0.16 ± 0.03	3.04 ± 0.36	0.21 ± 0.08
Total sugars				27.39	47.23	25.35	34.42	32.10	66.73	17.07	6.59	15.52	16.66
Sugar alcohols													
27	16.36	1729	Ribitol (5 TMS)	0.30 ± 0.48	0.01 ± 0.00	0.01 ± 0.01	0.01 ± 0.00	0.04 ± 0.04	(-)	0.02 ± 0.02	0.01 ± 0.00	0.02 ± 0.02	0.01 ± 0.00
28	16.49	1741	Unknown (5 TMS)	0.09 ± 0.02	0.50 ± 0.10	0.07 ± 0.03	0.07 ± 0.01	0.11 ± 0.04	0.03 ± 0.01	0.17 ± 0.03	0.16 ± 0.03	0.13 ± 0.02	0.16 ± 0.04
29	16.56	1746	Arabitol (5 TMS)	0.02 ± 0.01	0.39 ± 0.10	0.01 ± 0.01	0.01 ± 0.00	0.01 ± 0.01	0.01 ± 0.00	0.02 ± 0.00	0.04 ± 0.03	0.02 ± 0.02	0.03 ± 0.01
30	16.87	1772	meso-Erythritol (4 TMS)	0.59 ± 0.03	0.37 ± 0.09	0.48 ± 0.06	0.44 ± 0.06	0.46 ± 0.13	0.16 ± 0.03	0.64 ± 0.07	0.74 ± 0.08	0.65 ± 0.04	0.76 ± 0.14
39	17.81	1857	Pinitol (5 TMS)	0.07 ± 0.01	0.15 ± 0.16	0.15 ± 0.22	0.15 ± 0.18	6.11 ± 8.08	1.34 ± 0.36	0.06 ± 0.02	0.04 ± 0.00	0.05 ± 0.00	0.07 ± 0.04
45	18.87	1957	Sorbitol (6 TMS)	0.05 ± 0.02	0.27 ± 0.04	0.04 ± 0.03	0.05 ± 0.01	0.11 ± 0.07	0.03 ± 0.00	0.09 ± 0.01	0.09 ± 0.02	0.11 ± 0.03	0.06 ± 0.02
46	19.13	1982	Pinitol isomer (5 TMS)	0.23 ± 0.31	0.13 ± 0.14	0.43 ± 0.14	0.30 ± 0.35	0.08 ± 0.04	0.09 ± 0.05	0.23 ± 0.30	0.10 ± 0.03	0.16 ± 0.02	0.20 ± 0.17
49	20.49	2122	Myo-Inositol (6 TMS)	0.86 ± 0.08	0.52 ± 0.05	0.68 ± 0.35	0.56 ± 0.31	1.07 ± 0.24	0.58 ± 0.09	0.64 ± 0.18	0.51 ± 0.22	0.88 ± 0.22	0.55 ± 0.06
70	28	2963	Galactinol (9 TMS)	0.06 ± 0.02	0.94 ± 0.25	0.17 ± 0.04	0.30 ± 0.04	0.59 ± 0.22	1.45 ± 0.37	0.07 ± 0.01	0.14 ± 0.12	0.13 ± 0.01	0.26 ± 0.11
Total sugar alcohols				2.26	3.28	2.05	1.89	8.59	3.69	1.94	1.82	2.16	2.08
Total 100%				100	100	100	100	100	100	100	100	100	100

## Data Availability

Not applicable.

## Supplementary Materials

The following supporting information can be downloaded at: https://www.mdpi.com/article/10.3390/metabo13020163/s1, Figure S1. GC/MS chromatogram of the n-alkanes series (C20-C40) according to their retention indices (RI). Figure S2: GC/MS dataset based OPLS-DA score plot (A) derived from modelling silylated primary metabolites of all mature samples against all immature ones (*n* = 3). The respective loading S-plots (B) shows the covariance p[1] against the correlation p(cor)[1] of the variables of the discriminating component of the OPLS-DA model.Cut-off values of *p* = 0.001was used. Designated variables are highlighted and identifications are discussed in the text; Figure S3: GC/MS based OPLS-DA score plot (A) derived from modelling silylated primary metabolites of mature vs. immature *T. aestivum* (cv.*Gemeza 11*) *specimens* (*n* = 3). The respective loading S-plots (B) shows the covariance p[1] against the correlation p(cor)[1] of the variables of the discriminating component of the OPLS-DA model. Cut-off values of *p* = 0.09 was used. Designated variables are highlighted and identifications are discussed in the text; Figure S4: GC/MS based OPLS-DA score plot (A) derived from modelling silylated primary metabolites of mature vs. immature *V. faba* (cv. *Sakha 3*) *specimens* (*n* = 3). The respective loading S-plots (B) shows the covariance p[1] against the correlation p(cor)[1] of the variables of the discriminating component of the OPLS-DA model. Cut-off values of *p* = 0.06 was used. Designated variables are highlighted and identifications are discussed in the text; Figure S5: A bar graph showing diagrammatic values of the protein % (*w*/*w*) of both immature and mature seeds side by side. Each bar represents mean ± SE (*n* = 3); Table S1: A summary of the total protein assay results, showing the nitrogen (%*w*/*w*) of each sample, as well as the protein (*w*/*w*) after factor conversion. All results are expressed as mean ± SD, (*n* = 3).
