# Supplementary material for: Effect of Maturity Stage on Cereal and Leguminous Seeds’ Metabolome as Analyzed Using Gas Chromatography Mass-Spectrometry (GC-MS) and Chemometric Tools
Source: Metabolites. 2023 Jan 23;13(2):163. doi: 10.3390/metabo13020163 (PMC9960208; doi:10.3390/metabo13020163)
Supplement: Supplementary file 1 [file metabolites-13-00163-s001.zip › metabolites-2112803-supplementary.pdf]

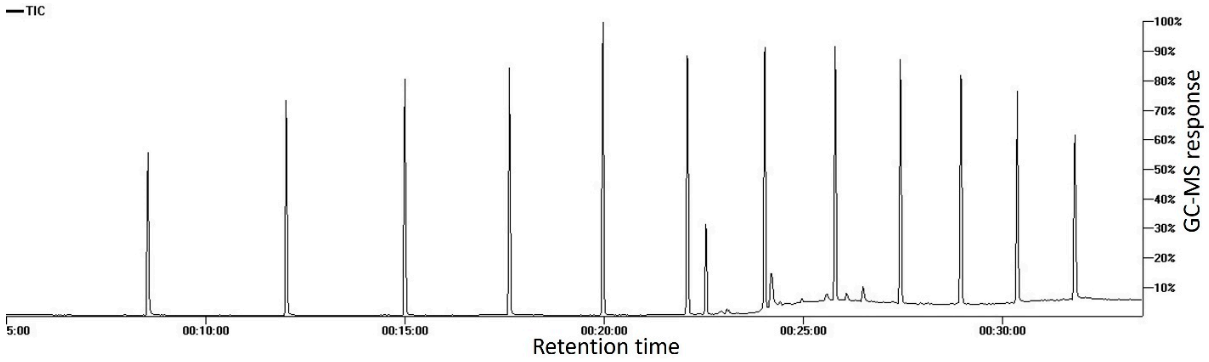

Figure S1. GC/MS chromatogram of the n-alkanes series (C20-C40) according to their retention indices (RI).

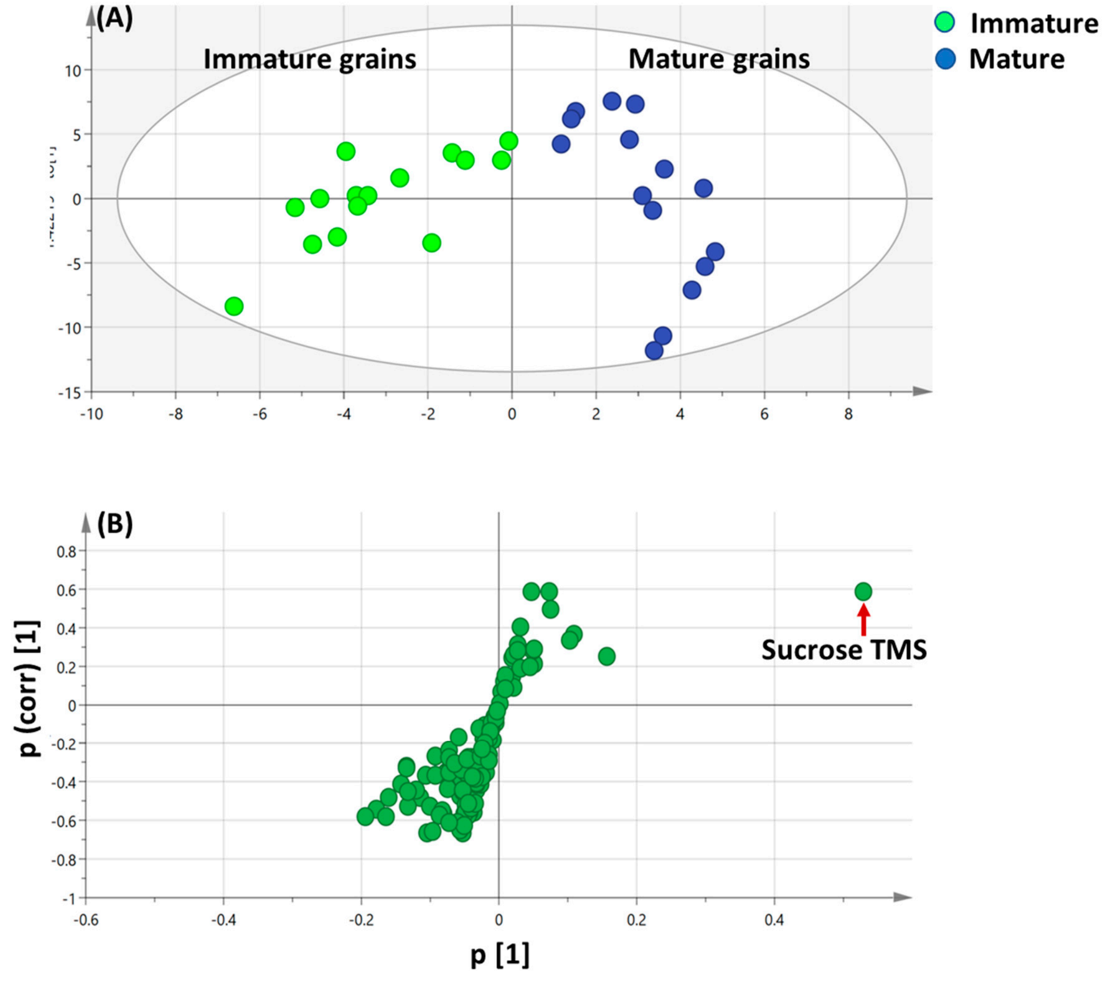

Figure S2. GC/MS dataset based OPLS-DA score plot (A) derived from modelling silylated primary metabolites of all mature samples against all immature ones (n = 3). The respective loading S-plots (B) shows the covariance p[1] against the correlation p(cor)[1] of the variables of the discriminating component of the OPLS-DA model.Cut-off values of p = 0.001was used. Designated variables are highlighted and identifications are discussed in the text.

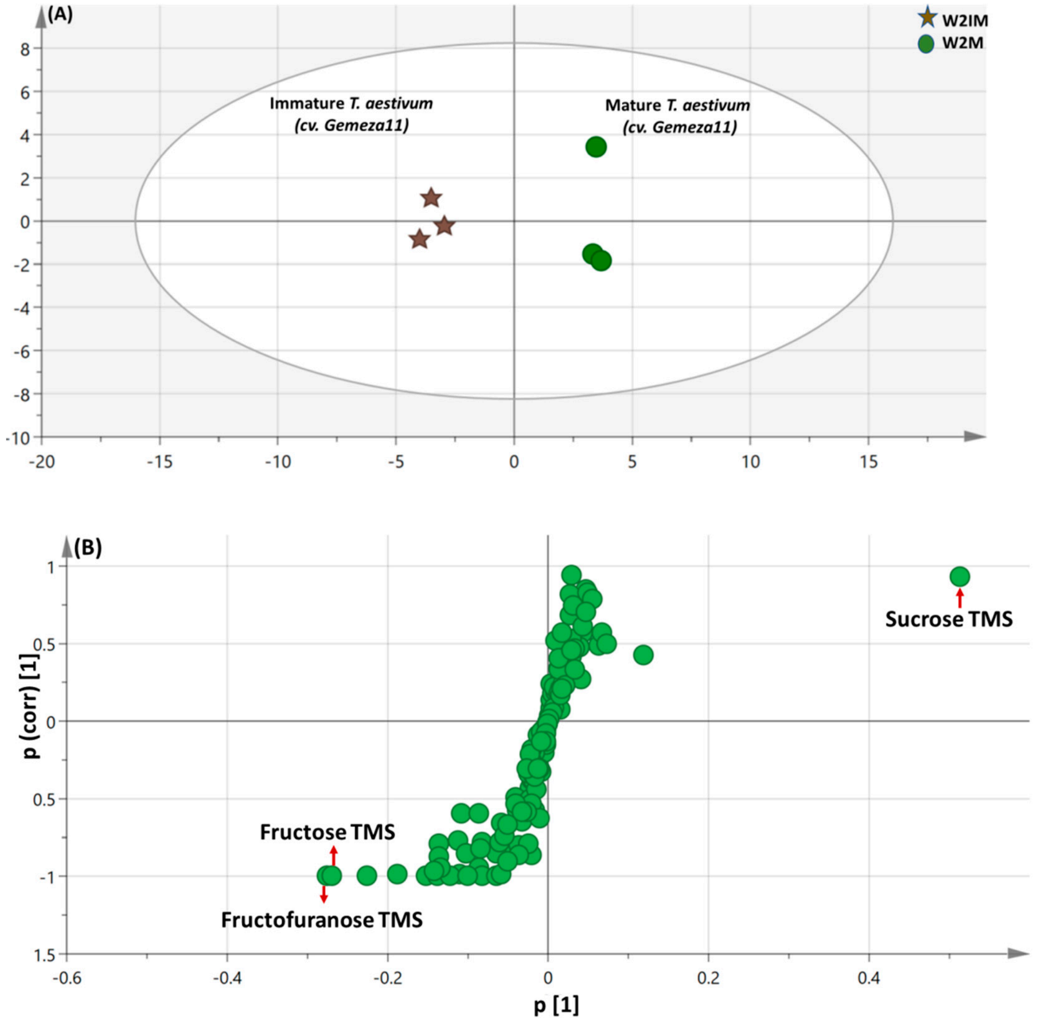

Figure S3. GC/MS based OPLS-DA score plot (A) derived from modelling silylated primary metabolites of mature vs. immature *T. aestivum (cv. Gemeza 11) specimens* (n = 3). The respective loading S-plots (B) shows the covariance p[1] against the correlation p(cor)[1] of the variables of the discriminating component of the OPLS-DA model. Cut-off values of p = 0.09 was used. Designated variables are highlighted and identifications are discussed in the text.

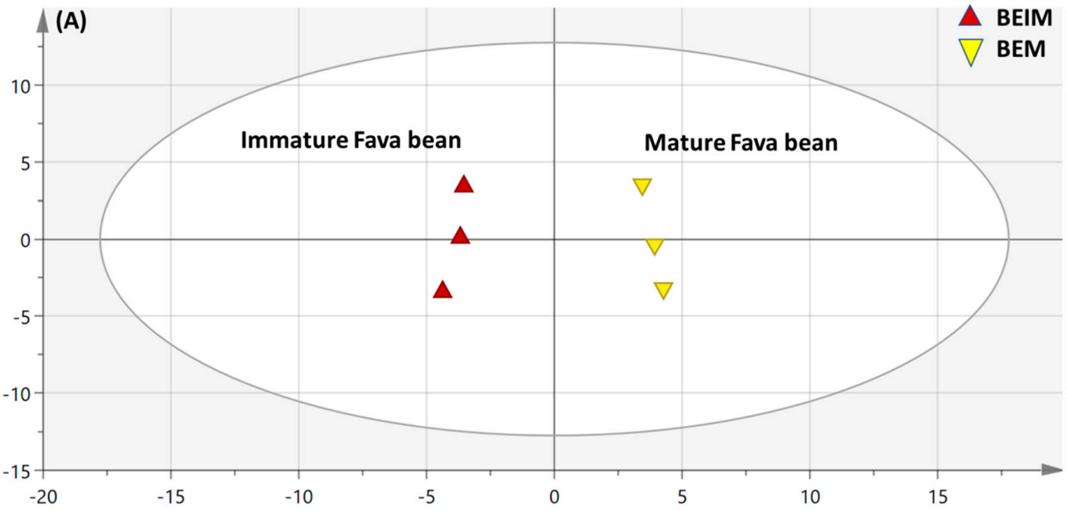

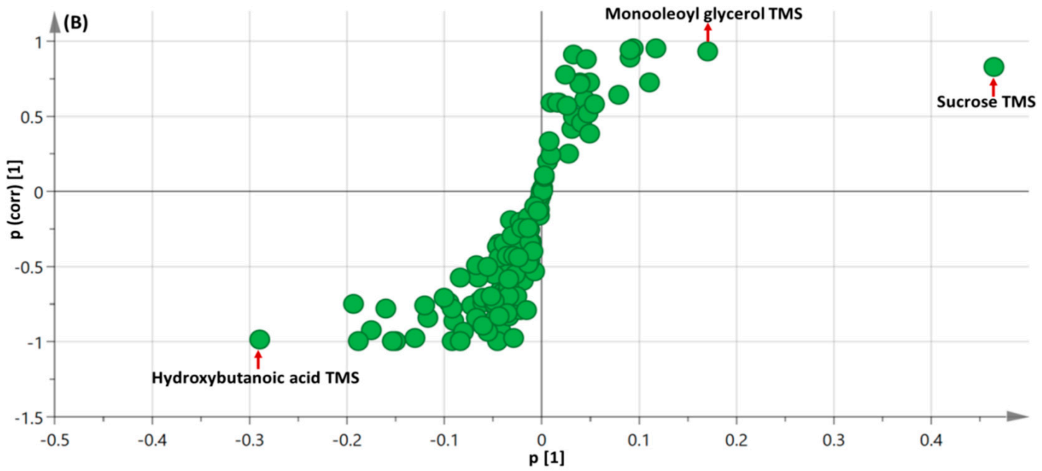

Figure S4. GC/MS based OPLS-DA score plot (A) derived from modelling silylated primary metabolites of mature vs. immature *V. faba* (*cv. Sakha 3*) *specimens* (n = 3). The respective loading S-plots (B) shows the covariance p[1] against the correlation p(cor)[1] of the variables of the discriminating component of the OPLS-DA model. Cut-off values of p = 0.06 was used. Designated variables are highlighted and identifications are discussed in the text.

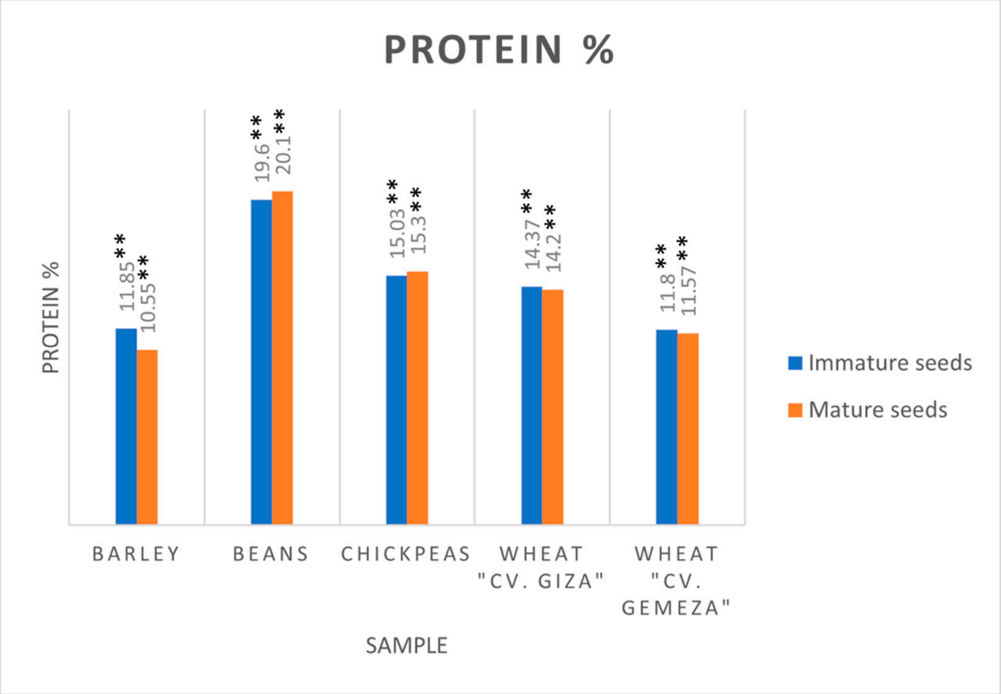

Figure S5. A bar graph showing diagrammatic values of the protein % (w/w) of both immature and mature seeds side by side. Each bar represents mean (n=3). **Statistically non-significant (*P* value > 0.05, one-way ANOVA).

Supplementary Table S1. A summary of the total protein assay results, showing the nitrogen (%w/w) of each sample, as well as the protein (w/w) after factor conversion. All results are expressed as mean ± SD, (n=3).

**Samples**	**N% (w/w)**	**Protein% (w/w)**
BIM	2.04±0.32	11.85±1.83 **
BM	1.82±0.16	10.55±0.93 **
BEIM	3.38±0.66	19.6±0.35 **
BEM	3.47±0.42	20.1±2.43 **
CIM	2.60±0.13	15.03±0.76 **
CM	2.63±0.04	15.3±0.2 **
W1IM	2.48±0.09	14.37±0.51 **
W1M	2.45±0.08	14.2±0.5 **
W2IM	2.04±0.02	11.8±0.1 **
W2M	1.99±0.03	11.57±0.21 **

**Statistically non-significant (*P* value > 0.05, one-way ANOVA).
